# Feather corticosterone reveals stress associated with dietary changes in a breeding seabird

**DOI:** 10.1002/ece3.1694

**Published:** 2015-09-07

**Authors:** Alexis Will, Yutaka Watanuki, Dale M. Kikuchi, Nobuhiko Sato, Motohiro Ito, Matt Callahan, Katherine Wynne‐Edwards, Scott Hatch, Kyle Elliott, Leslie Slater, Akinori Takahashi, Alexander Kitaysky

**Affiliations:** ^1^ Department of Biology and WildlifeInstitute of Arctic BiologyUniversity of Alaska Fairbanks311 N. Koyukuk Dr.FairbanksAlaska99775; ^2^ Graduate School of FisheriesHokkaido UniversityMinato‐cho 3‐1‐1Hakodate041‐8611Japan; ^3^ National Institute of Polar ResearchTachikawaTokyo190‐8518Japan; ^4^ Department of Polar ScienceSOKENDAITachikawaTokyo190‐8518Japan; ^5^ National Oceanic and Atmospheric AdministrationTed Stevens Research Institute17109 Pt. Lena Loop Rd.JuneauAlaska; ^6^ Faculty of Veterinary Medicine and Hotchkiss Brain InstituteUniversity of CalgaryHRIC 1B41 3330 Hospital Drive NWCalgaryAlbertaT2N 4N1Canada; ^7^ Institute for Seabird Research and Conservation12850 Mountain PlaceAnchorageAlaska99516; ^8^ Department of Natural Resource SciencesMcGill UniversitySte Anne de BellevuePQ H9X 3V9MontrealQuebecCanada; ^9^ Alaska Maritime National Wildlife RefugeUnited States Fish and Wildlife Service95 Sterling HwySuite 1HomerAlaska99603

**Keywords:** Diet composition, foraging behavior, junk‐food hypothesis, nutritional stress, rhinoceros auklet

## Abstract

Changes in climate and anthropogenic pressures might affect the composition and abundance of forage fish in the world's oceans. The junk‐food hypothesis posits that dietary shifts that affect the quality (e.g., energy content) of food available to marine predators may impact their physiological state and consequently affect their fitness. Previously, we experimentally validated that deposition of the adrenocortical hormone, corticosterone, in feathers is a sensitive measure of nutritional stress in seabirds. Here, we use this method to examine how changes in diet composition and prey quality affect the nutritional status of free‐living rhinoceros auklets (*Cerorhinca monocerata*). Our study sites included the following: Teuri Is. Japan, Middleton Is. central Gulf of Alaska, and St. Lazaria Is. Southeast Alaska. In 2012 and 2013, we collected “bill loads” delivered by parents to feed their chicks (*n* = 758) to document dietary changes. We deployed time–depth–temperature recorders on breeding adults (*n* = 47) to evaluate whether changes in prey coincided with changes in foraging behavior. We measured concentrations of corticosterone in fledgling (*n* = 71) and adult breeders' (*n* = 82) feathers to determine how birds were affected by foraging conditions. We found that seasonal changes in diet composition occurred on each colony, adults dove deeper and engaged in longer foraging bouts when capturing larger prey and that chicks had higher concentrations of corticosterone in their feathers when adults brought back smaller and/or lower energy prey. Corticosterone levels in feathers of fledglings (grown during the breeding season) and those in feathers of adult breeders (grown during the postbreeding season) were positively correlated, indicating possible carryover effects. These results suggest that seabirds might experience increased levels of nutritional stress associated with moderate dietary changes and that physiological responses to changes in prey composition should be considered when evaluating the effect of prey quality on marine predators.

## Introduction

Ecological changes such as increased competition (e.g., Svanbäck and Bolnick 2007), disease (e.g., Moleón et al. 2009), or fluctuations in the availability and abundance of food (e.g., Jackson and Rundle 2008; Zhou et al. 2015) can result in short‐term changes in the composition and quality (e.g., energy content) of an animal's diet. According to the junk‐food hypothesis, changes in the quality of prey can affect a marine animal's ability to survive and reproduce (Alverson 1992). Climate change and anthropogenic pressures might induce shortages in the availability of energy‐rich prey to marine predators, such as seabirds (Kitaysky et al. 2006; Essingtona et al. 2015); yet studies of how prey quality affects reproductive performance in seabirds provide mixed support for the junk‐food hypothesis. Some studies find no evidence that changes in prey quality affect reproductive output (Jodice et al. 2006; Kadin et al. 2012; Hjernquist and Hjernquist, 2010). Others clearly demonstrate that low‐quality prey, such as fisheries offal (Grémillet et al. 2008), or changes in the energy content, availability, or abundance of a preferred energy‐rich prey species (Wanless et al. 2007; Dorresteijn et al. 2012; Barrett et al. 2015), negatively impact reproductive performance and adult nutritional status (Dorresteijn et al. 2012; Barrett et al. 2015).

The impact of prey quality (i.e., total caloric content which is often driven by lipid richness) and quantity may be best examined on a continuum, with switches between prey of equal quality on one end and changes between high‐ and low‐quality prey at the other. With this approach, we expect that a switch from high‐ to low‐quality prey would only impact reproductive performance and/or survival in cases where prey quality changed substantially and could not be counteracted by increasing the quantity of the low‐quality food. If switches occur among prey of relatively equal energy value, overall reproductive performance may not vary, but the physiology and behavior of individuals may be affected.

**Figure 1:**
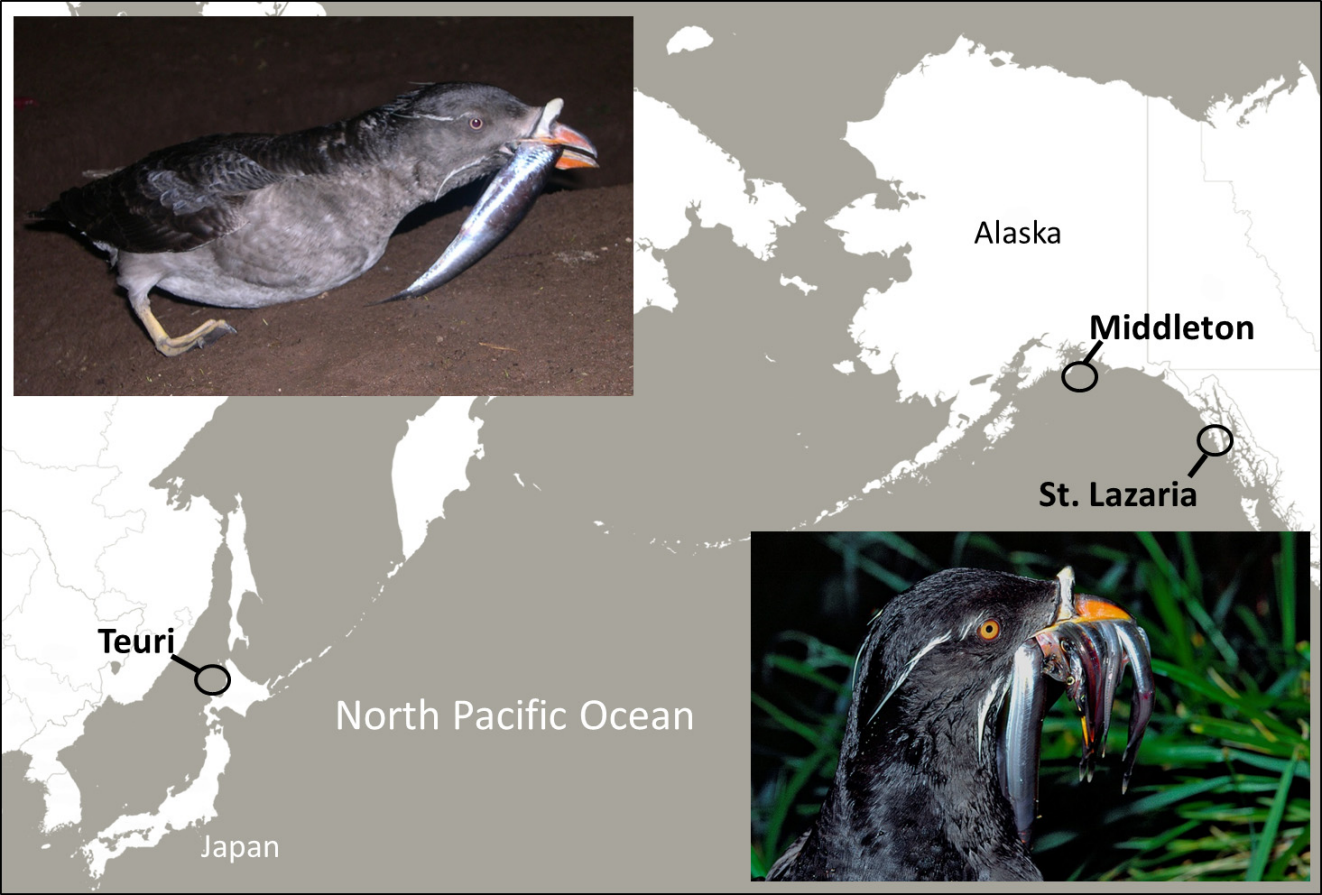
The rhinoceros auklet and field sites. Photographs are of rhinoceros auklets returning to their colonies at night with a “bill load” of fish for their chicks on Teuri Is. by Motohiro Ito (top left) and on Chowiet Is., Gulf of Alaska, by Nikolai Konyukhov (bottom right). Base map from Stamen Maps.

Many seabird species (e.g., see Kitaysky et al. 1999; Benowitz‐Fredericks et al. 2008; Rector et al. 2012) respond to nutritional stress with increased levels of the avian stress hormone, corticosterone (CORT). Researchers use concentrations of CORT as a relative measure to infer the nutritional stress that birds incur. Nutritional stress occurs when individuals experience a negative balance between their energy needs and the energy that is available (Kitaysky et al. 2001b). The increased secretion of CORT in response to a decrease in energy intake enables an individual to survive the event (Kitaysky et al. 2001a, 2003), but stress incurred during the event can take a toll, impacting their ability to survive and reproduce in the future (Kitaysky et al. 2010). To test how changes in prey composition and quality affect seabirds, we examined the chick diets, adult behavior, and the stress status of chick and adult rhinoceros auklets (*Cerorhinca monocerata,* hereafter RHAU, Fig. 1), a coastal, pursuit‐diving piscivore that breeds in the North Pacific.

First, we examined how chick diets changed over the course of the breeding season. Then, we focused our investigation on the relationship between diet and adult foraging behavior, which may mediate the impact of diet changes on both adults and chicks. In cases of mild or temporary food shortages, breeding seabirds may adjust their foraging behavior (e.g., Burke and Montevecchi 2009; Harding et al. 2009, 2013) by increasing dive frequency (Ronconi and Burger 2008; Hedd et al. 2009), the number of foraging bouts, and/or the time spent underwater in pursuit of prey (Karnovsky et al. 2011).

Finally, we examined the concurrent physiological status of chicks and the postreproductive status of adults. We measured CORT in feather tissues to assess whether changes in diet composition affected physiology. Previously, we have shown that experimentally induced nutritional stress is associated with higher concentrations of CORT in RHAU fledgling's feathers (Will et al. 2014). This endocrine technique allows for a one‐time handling of fledglings to obtain an integrated measure of stress physiology (Bortolotti et al. 2008) during the growth of the sampled feather (up to 48 days, Gaston and Dechesne 1996). We predicted that changes from high‐ to low‐quality prey would affect parental foraging behavior and result in elevated CORT concentrations in fledgling and adult feathers.

## Methods

### Study sites

We chose three colonies which differ dramatically in their oceanography and prey dynamics. Fieldwork occurred in 2012, 2013, and 2014 (a follow‐up year to collect adult feathers) on St. Lazaria Island (56°59′N 135°42′W), 22 km west of Sitka, Alaska at the entrance to Sitka Sound in the Eastern Gulf of Alaska (Fig. 1) and home to ~2000 pairs of breeding RHAU which prey heavily upon capelin (*Mallotus villosus)* and Pacific sand lance (*Ammodytes hexapterus*) (Slater and Byrd 2009; Hovis and Slater 2012). Middleton Island (59°26′N 146°19′W), in the northern Gulf of Alaska (Fig. 1), has 7500 to 10,000 breeding pairs (S. Hatch pers. comm.) that feed predominantly on capelin (Hatch 2013). Teuri Island (44°25′N: 141°18′E), in Northern Japan (Fig. 1), where the world's largest population of RHAUs (~300,000 breeding pairs) provision their young primarily with Japanese anchovy (*Engraulis japonicus*) (Watanuki et al. 2009).

### Study species

The RHAU is a nocturnal, burrow‐nesting seabird, with biparental care of a single chick. Chicks receive at most two meals per night (one per parent) delivered as a “bill load,” a collection of whole fish which adults carry crosswise in their bill (Fig. 1, Gaston and Dechesne 1996). Across much of their range RHAU reproductive success correlates positively with the presence of a particular prey species in their chick's diet (Hedd et al. 2006; Watanuki and Ito 2012). Experimental work indicates that RHAU chicks preferentially allocate limited resources to the growth and development of organs and skeletal structures needed for fledging (Takenaka et al. 2005; Hirose et al. 2012) and retain a typical physiological response (increased CORT) to reduced caloric intake (Sears and Hatch 2008; Will et al. 2014). Chick diet composition closely resembles parental diet during the chick‐rearing period (Ito et al. 2009; Hipfner et al. 2013).

### Chick diet

We collected bill loads from parents throughout the chick‐rearing period (late June–August St. Lazaria and Middleton, and late May–July Teuri) to assess changes in diet composition. While bill loads were collected during capture and recapture of adults outfitted with time‐depth‐temperature recorders (TDR), the greater portion of samples were collected from birds not participating in that aspect of the study. These birds were intercepted on the ground as they returned to the colony at dusk. Fish were identified to species or, for greenling (*Hexigrammidae* spp.), to genus, individually weighed, measured, and, on St. Lazaria, frozen in the field for postseason energy content analysis. For both 2012 and 2013, we sampled eight capelin, greenling, Pacific herring (*Clupea pallasii*), Pacific sand lance, and salmon species (*Oncorhynchus* spp.), for a total of 16 fish per species, except herring which was nearly absent from 2012 bill loads. Fish sampled were of average length and collected throughout the study period. Individual fish were homogenized using a mortar and pestle and dried with a Leco Thermogravimetric Analyzer (St. Joseph, Michigan, USA), which also measured moisture content. Dried samples were crushed into powder and pressed into 150–200 mg pellets for bomb calorimetry. Energy density (kJ g^−1^ dry) was determined using a Parr 6725 semimicro calorimeter (Moline, IL, USA); see Siddon et al. (2013) for full method details. Energy content for fish from other islands was derived from previously published values (van Pelt et al. 1997; Takahashi et al. 2001).

Bill load energy content was determined by multiplying the energy content (wet mass: kJ g^−1^) for each fish species by the field‐recorded mass of the individual fish and the products summed for all fish in each complete bill load.

### Adult foraging behavior

On St. Lazaria breeding adult RHAUs were captured at night while entering their burrows. G5 Cefas (Cefas Technologies Ltd., Lowestoft, Suffolk, UK) TDRs were mounted on a size 5 plastic tarsal band (Pro Touch Engraving, Saskatoon, SK, Canada) using 5‐min epoxy and two zip ties. The entire assembly weighed ~ 4 g, < 1% of the average St. Lazaria RHAU's body mass. Loggers were preprogrammed to collect data for two to five days and were deployed throughout the chick‐rearing period beginning in early July and continuing through mid‐August. Recapture effort began the day before tags finished logging and, in some cases, continued until the end of the season. We recovered 50% and 83% of tags in 2012 and 2013, respectively.

On Teuri, adults were captured at night in their burrows. Birds were outfitted with accelerometers (ORI‐D3GT: *ϕ*12 × 45 mm, 9 g, Little Leonardo Corp., Tokyo, Japan) that also recorded temperature and diving depth. Loggers recorded data for one or two days and were deployed during a one‐week period midway through the breeding season (for details see Kikuchi et al. 2015).

No dive data are available for Middleton. TDRs (LAT290, Lotek Marine Technology, St. John's, Newfoundland, Canada) were deployed, but the tag settings made the data incomparable to data from St. Lazaria and Teuri.

Adults were sexed genetically using erythrocyte‐extracted DNA and amplification of the CHD1 gene (Griffiths et al. 1998) and morphologically sexed using a discriminant equation (96% probability of correct differentiation) based on bill measurements (Niizuma et al. 1999).

### Chick and adult nutritional stress

We captured chicks in the act of fledging (parents and age not known, range 42–58 days old; Gaston and Dechesne 1996) and sampled their first primary (P1, clipped at the base), grown from day 10 to 49 posthatch (Will et al. 2014). Feathers were prepared according to Will et al. (2014) and Bortolotti et al. (2008). Briefly, the entire feather was divided into three 20‐mm segments to be analyzed for CORT (hereafter, fCORT) separately for measures of early, middle, and late feather growth stress status. Each segment was minced and then extracted in 7 mL methanol (HPLC‐grade, Fisher Scientific, Waltham, MA). Feather segments were analyzed in a radioimmunoassay (Wingfield and Farner 1975) using a Sigma‐Aldrich antibody (C 8784, Saint Louis, MO), intra‐assay CV < 1% and interassay (3 assays) CV 1.5%. To control for loss of CORT during the extraction process, 2,000 cpm of H^3^‐labeled CORT (PerkinElmer NET399, Boston, MA) was added to each sample and final fCORT titers were adjusted for % recovery (mean = 89%). Assay results were normalized by converting to units of pg mm^−1^ (Bortolotti et al. 2008). Results from 2012 appeared previously in Will et al. (2014) as part of a validation study.

The RHAU undergoes a sudden postreproductive molt of its primary feathers (Gaston and Dechesne 1996); thus, in any given breeding season, it is possible that adult fCORT reflects physiological status at the end of the previous breeding season. We measured carryover effects of the breeding to postreproductive season by recapturing breeding individuals the subsequent year (capture and handling during the previous year had no effect on fCORT concentrations, A. Will unpubl. data) and sampling the first primary (all colonies, all years except Teuri, 2013, when the 10th primary was sampled, fCORT concentrations were within the ranges measured in P1). On St. Lazaria, feathers were collected from TDR birds caught in 2013 and again in 2014; however, on Teuri and Middleton, samples were collected from untagged breeding adults. Feather growth rate for adult RHAU is not well known, but assuming a similarity with their close relative, the tufted puffin, *Fratercula cirrhata* (Thompson and Kitaysky 2004), we estimate that the adult feather segments we analyzed were grown during ~10 days at the start of the postreproductive molt. Feathers were prepared and analyzed following the same protocol as for chicks with the following exceptions: only 25 mm of the tip was sampled for analysis, washed with deionized water and isopropanol (HPLC‐grade, Sigma‐Aldrich, St. Louis, Missouri, USA) to remove dirt and oils, and extracted intact. Intra‐assay CV <1%, interassay (3 assays) CV 5.9%, and final fCORT titers were adjusted for % recovery (mean = 95.8%).

The Sigma antibody used in our assay has a high affinity for corticosterone but also demonstrates some cross‐reactivity with other steroids; therefore, we also verified the presence of the corticosterone molecule in both chick and adult primary feather tissues using high‐performance liquid chromatography tandem mass spectrometry (Appendix S1, Koren et al. 2012).

### Statistical analysis

We divided the breeding season into “Early” and “Late” periods to evaluate intra‐annual changes in chick diet and adult foraging behavior. Due to the timing of when samples and adult behaviors were recorded, we could not further divide these variables into three intervals to match the fledgling feather segments because some intervals would have no data points. These periods were determined using the average length of primary feathers collected from fledglings for a given island to back‐calculate from the average fledgling capture date. We assumed primaries grew at 2.2 mm/day (Will et al. 2014), then divided the total days of the feather growth period in half. We subtracted this number from the average fledgling capture date to arrive at the boundary of “Early” and “Late” chick rearing.

Adult Foraging Behavior – TDR data were first processed in IGOR Pro (WaveMetrics 2008) following Ito et al. (2010) to summarize the duration (sec) and depth (m) of each recorded dive. We calculated a bout‐ending criteria following Sibly et al. (1990) for each colony and each year. Using this value, we assigned dives to foraging bouts and calculated the number of dives per bout, how long (sec) the bout lasted (bout duration), and the average depth (m) of dives in a bout (average bout depth) for each bird. We also calculated the number of bouts that occurred each day and the total time (sec) a bird spent underwater in a day. All dive parameters, except for time underwater, were severely right skewed; therefore, we used general linear mixed models (*lme4* package in R) with bird as a random factor and either a Poisson or, to account for overdispersion in the data, a negative binomial distribution. All models were evaluated with a goodness‐of‐fit test and returned a *χ* ^2^ = 1. Dive depth was converted to whole numbers, and time underwater was square‐root transformed to satisfy assumptions of count values (Poisson) and a normal distribution (linear model), respectively. Because we were primarily interested in overall differences in diving behavior between years, we excluded sex from our final models due to small sample sizes in both years on Teuri. However, before exclusion, we ran the models with sex to verify that it did not alter the results. Finally, we were unable to run a model comparing diving behavior to diets because the number of TDR birds with associated diets was too low and restricted to St. Lazaria.

Chick and Adult Nutritional Stress Status – fCORT concentrations (pg mm^−1^) were log‐transformed to meet assumptions of normality and were significantly (mixed model with fledgling as a random factor; effect of feather segment mass, g: *F*_1,143_ = 76.45, *P* < 0.0001) and positively (parameter estimate: 739.93 ± 84.62) correlated with feather segment mass. Therefore we detrended fCORT values using residuals calculated from the best fit linear model of log‐transformed feather mass (log_10_(g)) and log‐transformed fCORT concentrations (log_10_(pg mm^−1^)), for chicks and adults separately. We use these detrended values in all figures and statistical analyses. Previously, we showed that sex does not affect fledgling's fCORT (Will et al. 2014), nor did we find a significant difference between fCORT concentrations in adult male and female feather tissues (*t*_33_ = 0.18, *P* = 0.43), so we did not include sex in our analysis.

All analyses were completed in R (version 3.1.2, Vienna, Austria 2014). For each analysis, we tested and report the full model.

## Results

### Chick diet

Bill load energy content attributed to a preferred prey: capelin on St. Lazaria and Middleton, and Japanese anchovy on Teuri (Table 1), changed on all three islands in both years. On Middleton and St. Lazaria, when birds did not return with capelin, they delivered species whose energy content was similar, such as herring and sand lance. Whereas when Japanese anchovy were not available on Teuri, adults made up the difference with lower quality prey (Tables 1 and 2). Similarly, on Middleton and St. Lazaria, pink salmon and greenling, respectively, constituted >10% of the diet at times and were not has high in caloric value as the other three primary prey species (capelin, sand lance, and herring) at those locations.

**Table 1:** Prey composition of RHAU bill loads. Percent of energy attributed to prey species in RHAU bill loads collected on St. Lazaria, Middleton, and Teuri Islands. “Early” refers to the first half of the chick‐rearing period, and “Late,” to the second half (see Methods for details). The number of bill loads collected in each period is listed in parentheses and the primary prey species for each colony is highlighted in bold

	2012		2013	
	% Early	% Late	% Early	% Late
**St. Lazaria**	(*n* = 38)	(*n* = 41)	(*n* = 50)	(*n* = 47)
***Capelin***	40.57	54.48	44.97	11.42
*Pacific Sandlance (A. hexapterus)*	50.58	30.1	23.67	26.56
*Pacific Herring*	3.4	3.27	12.95	50.36
*Greenling* spp.	2.53	8.28	16.98	9.33
*Salmon* spp.	2.92	3.87	1.43	2.33
**Middleton**	(*n* = 99)	(*n* = 113)	(*n* = 109)	(*n* = 82)
***Capelin***	94.7	50	82.74	36.22
*Pink Salmon*	2.4	29.9	0.68	15.82
*Pacific Herring*	0.06	7	15.78	42
*Greenling* spp.	1.8	5.7	0.32	0.89
*Chum Salmon*	0.3	0	0	3.38
*Other*	0.74	7.4	0.48	1.69
**Teuri**	(*n* = 30)	(*n* = 59)	(*n* = 50)	(*n* = 40)
***Japanese Anchovy***	70	99	99.4	86.1
*Japanese Greenling*	15.6	0	0.2	0
*Sandlance +1 (A. personatus)*	8.6	0	0.4	4.2
*Sandlance 0 (A. personatus)*	0	1	0	8.4
*Other*	5.8	0	0	1.3

**Table 2:** Energy content of RHAU prey species. Energy content is measured as kJ g^−1^ wet mass and was derived from (1) analysis of prey from St. Lazaria RHAU bill loads in 2012 and 2013 (see Methods) and (2) “Published,” Takahashi et al. (2001). Means are ± SE. Differences in Pacific sand lance (*A. hexapterus*) energy content were largely driven by fish size/age class, RHAU adults delivered age‐0 sand lance in 2012, and in 2013, they delivered age‐1+

	Energy content						
	2012	*n*	2013	*n*	*t*‐test	Published	*n*
*Capelin*	6.96 ± 0.21	8	6.97 ± 0.32	8	0.48		
*Pacific Sand lance (A. hexapterus)*	6.39 ± 0.22	8	6.96 ± 0.20	8	0.07		
*Pacific Herring*			5.97 ± 0.13	8			
*Greenling* spp.	5.75 ± 0.13	8	5.63 ± 0.20	8	0.3		
*Pink Salmon*	4.53 ± 0.12	5	4.95 ± 0.17	3	0.05		
*Chum Salmon*	4.38 ± 0.15	3	4.55 ± 0.18	5	0.27		
*Japanese Anchovy*						6.29 ± 1.47	6
*Japanese Greenling*						4.78 ± 0.63	6
*Sand lance +1 (A. personatus)*						5.47 ± 1.93	3
*Sand lance 0 (A. personatus)*						3.78 ± 0.44	3

### Adult behavior

Adult diving behavior varied significantly both between colonies and between years. Bout duration and bout depth were greater for Teuri birds than for those on St. Lazaria, but dives per bout and bouts per day were fewer (see Table 3). Birds on both colonies had more foraging bouts per day and spent more time underwater in 2013 than in 2012.

**Table 3:** Summary of diving parameters for chick‐rearing RHAU on St. Lazaria Island (2012, 11 birds and 36 bird days; 2013, 20 birds and 78 bird days) and Teuri Island (2012, 8 birds and 8 bird days; 2013, 8 birds and 11.5 bird days). Sample sizes (*n*) appear in parentheses above the means ± SE

	St. Lazaria		Teuri		Model	*P*‐value		
	2012	2013	2012	2013		Year	Colony	Year*Colony
Bout‐based Parameters	(*n* = 878)	(*n* = 2779)	(*n* = 140)	(*n* = 267)				
Dives per bout	11.09 ± 0.60	9.76 ± 0.34	9.02 ± 0.84	8.04 ± 0.67	GLMM negative binomial	0.38	0.25	0.89
Bout duration (sec)	374.09 ± 23.25	391.57 ± 13.83	618.82 ± 66.66	593.37 ± 48.77	GLMM negative binomial	0.63	0.04	0.89
Average bout depth (m)	4.42 ± 0.15	4.39 ± 0.10	13.69 ± 0.91	15.54 ± 0.70	GLMM Poisson	0.84	<0.001	0.62
Bird‐based Parameters	(*n* = 36)	(*n* = 78)	(*n* = 8)	(*n* = 11.5)				
Bouts per day	24.39 ± 1.19	35.63 ± 1.43	17.50 ± 2.14	23.75 ± 2.32	LME	<0.001	0.003	0.41
Time Underwater per day (√sec)	74.49 ± 3.17	96.24 ± 2.68	84.03 ± 9.19	101.10 ± 7.06	LME	0.003	0.54	0.77

On St. Lazaria in 2012, tagged adult RHAUs more often skipped returning to the colony at least one night during tag deployment compared to 2013 (Fisher's exact test *P* = 0.03; 2012 proportion that skipped = 0.44, *n* = 9; 2013 proportion that skipped = 0.06, *n* = 18).

### Chick and adult nutritional stress status

**Figure 2:**
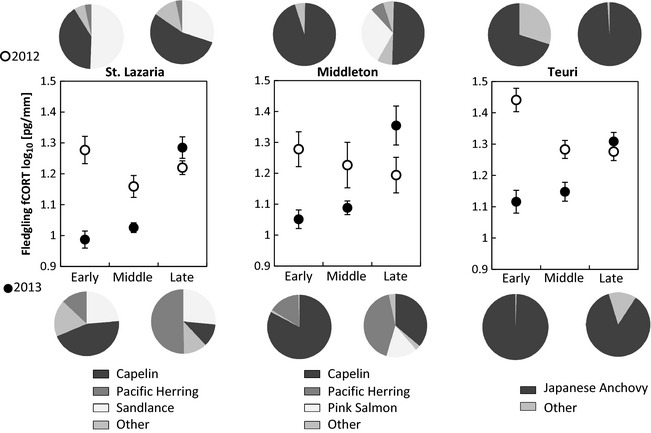
Temporal and spatial changes in diet composition and nutritional stress incurred by RHAU chicks. Log‐transformed fCORT concentrations measured in primaries of free‐living RHAU fledglings on St Lazaria (2012 *n* = 13; 2013 *n* = 14), Middleton (2012 *n* = 6; 2013 *n* = 7), and Teuri (2012 *n* = 15; 2013 *n* = 16) islands. Feather segments: Early = tip, Middle = middle, and Late = base of the feather ± SE. The pie charts illustrate bill load composition delivered during the first and second half of the breeding season. Proportions are of total energy delivered (kJ g^−1^, wet mass) per prey type.

In general, RHAU chicks experienced higher nutritional stress in 2012 than in 2013 on all colonies (mixed model fixed factor, year: parameter estimate = −0.46, *F*_1,66_ = 34.067, *P* ≤ 0.0001, Fig. 2). Chicks reared on different colonies had different fCORT concentrations (fixed factor, colony: *F*_2,66_ = 10.282, *P* = 0.0001). Nutritional stress changed significantly over the course of the chick‐rearing period in both years (fixed factor, feather segment: Fig. 2, *F*_2,138_ = 16.43, *P* = 0.0001). However, fCORT concentration within a season decreased in 2012 and increased in 2013 (interaction term, year*feather segment: *F*_1,138 _= 89.96, *P *≤ 0.0001). While this pattern occurred on all three colonies, intercolony differences in fCORT were still detectable within each feather segment (interaction term colony*feather segment: *F*_2,138_ = 3.512, *P* = 0.032). All other variables did not explain a significant proportion of the variability in the fCORT concentrations (interaction terms colony*year: *F*_2,66_ = 0.89, *P* = 0.42, colony*year*feather segment: *F*_2,138 _= 0.05, *P* = 0.95).

**Figure 3:**
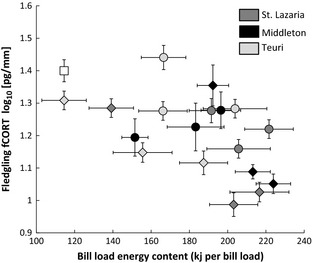
Bill load energy content and fCORT. Mean bill load energy (± SE) in diet samples collected at St. Lazaria, Middleton, and Teuri during early, middle, and late feather growth periods in 2012 (circles) and 2013 (diamonds). We compared these colony‐wide values to the average fCORT concentrations (± SE) of feather segments sampled from fledglings. (□) is the mean (± SE) fCORT concentration of captive‐reared chicks on a restricted diet (239 kJ day^−1^ or ~114 kJ per “parent”; Will et al. 2014) and is shown for comparative purposes.

Bill load energy content did not differ between years (ANOVA, year: *F*_1,10 _= 0.21, *P* = 0.65) but was different between colonies (ANOVA, colony: *F*_2,10_ = 3.99, *P* = 0.05). The seasonal pattern of bill load energy content among colonies tended to differ between years (ANOVA, year*colony: *F*_2,10_ = 3.66, *P* = 0.06) and within years (ANOVA, year*segment: *F*_1,10_ = 4.55, *P* = 0.06). Bill load mass was correlated to bill load energy content (simple linear regression: adjusted *R* ^2 ^= 0.8, *P* ≤ 0.001). However, fCORT by feather growth period (early, middle, late) corresponded more to changes in bill load energy content (mixed model, feather segment as a random factor: *F*_1,11_ = 7.24, *P* = 0.02, Fig. 3) than to changes in bill load mass (mixed model: *F*_1,11_ = 3.82, *P* = 0.08).

**Figure 4:**
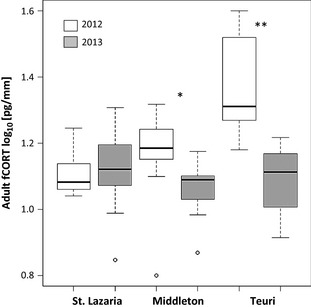
Feather CORT concentrations in adult feather tips. Median fCORT concentrations (solid line) shown in relation to the distribution of values for adults sampled on St. Lazaria (2012 *n* = 17; 2013 *n* = 19), Middleton (2012 *n* = 14; 2013 *n* = 15), and Teuri (2012 *n* = 7; 2013 *n* = 10). The distribution of adult fCORT concentrations is illustrated with boxes (middle 50%) ± whiskers (the outer 25%), and open circle “outliers” (no values were excluded from analysis). 2012 and 2013 refer to the year in which the feathers were grown; birds were sampled during the following breeding season. *P < 0.01, **P < 0.001.

**Figure 5:**
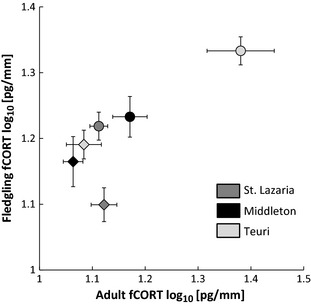
Corticosterone concentrations in postreproductive adult feathers correlate with CORT concentrations in fledgling feathers. Plotted are the average CORT concentrations measured in the tip (25 mm) of adult feathers and 20 mm segments of fledgling feathers for St. Lazaria, Middleton, and Teuri Islands for the 2012 (circles) and 2013 (diamonds) postreproductive (adults) and breeding (fledglings) seasons. Error bars are ± SE of the mean, adjusted *R* ^2 ^= 0.58, *P* = 0.048.

In general, adults had lower fCORT concentrations in feathers grown after the 2013 breeding season than those grown after the 2012 season (ANOVA, year: *F*_1,76_ = 15.35, *P* = 0.0002, Fig. 4). However, changes in fCORT between years was not the same on all colonies (ANOVA, year*colony: *F*_2,76_ = 12.45, *P* < 0.0001) because there was no difference in adult fCORT between years on St. Lazaria (Fig. 4). Feather CORT concentrations in adult feather tissues were positively correlated with fledgling fCORT concentrations on all three colonies in both years (Fig. 5).

## Discussion

We examined how changes in diet during reproduction affected a piscivorous seabird breeding on three spatially distant colonies in the North Pacific. We found that intra‐annual changes in diet occurred on all three of our focal colonies and that chick nutritional status (fCORT) and adult foraging behavior changed in tandem with prey species composition. Shifts in diet corresponded to changes in fCORT concentration in fledglings, which were mirrored in CORT concentrations in adult feathers grown after the breeding season. We acknowledge that the feathers used for CORT analysis may represent disproportionately high‐quality chicks and adults that were able to survive to fledging age (chicks) and through the winter (adults). However, these individuals did exhibit changes in fCORT concentration both seasonally and interannually; thus, it can be concluded that if our sampling were biased, the rest of the population may have been affected more severely by the diet changes we observed. Below, we discuss one possible interpretation of these results.

When searching for food, predators may optimize prey mass, energy content, lipid content, and/or macronutrient availability (Kohl et al. 2015) depending on environmental conditions and an individual's needs (e.g., in mammals see Gende et al. 2005). However, if prey quality is low, a predator may not have many optimal options. For example, in the North Sea, common murres (*Uria aalge*) experienced reproductive failure when adults fed copious amounts of seabream (*Sprattus sprattus*) to their chicks when sand eels (*Ammodytes marinus*) were unavailable (Wanless et al. 2005). Such differences in energy content may be driven by lipid content, which is often correlated with the total energy in forage fish species (van Pelt et al. 1997). For growing seabirds, lipids are critical to support growth and development; when absent, nestlings experience nutritional stress (Kitaysky et al. 2006; Romano et al. 2006). On Teuri, we found that even a small reduction in the proportion of Japanese anchovy in RHAU chick diets corresponded to an increase in fCORT, suggesting that the moderate differences in prey energy content (Table 2) may have been compounded by differences in lipid content (Takahashi et al. 2001). While the quantity of fish delivered is often correlated with energy content, we found that bill load energy content was more strongly correlated with changes in fledgling fCORT concentrations.

On Middleton and St. Lazaria, changes in diet composition between prey species equivalent in energy content also corresponded to changes in chick nutritional status (Fig. 2). In animals with parental care, adults act as an interface between foraging conditions and their young and must balance their own energy intake while still providing for their offspring. This may result in adjustments to their daily delivery rate when prey becomes more or less available (Welham and Beauchamp 1997); a response observed in many seabird species (e.g., Hamer and Hill 1993; Schrimpf et al. 2012; Kidawa et al. 2015). Experimental work on Teuri indicated that reduced meal frequency negatively affects chick body condition (Takenaka et al. 2005). Thus, meal delivery rate may explain some of the interannual variabilities in chick nutritional stress on the Alaskan colonies. On St. Lazaria, adult's colony attendance, a proxy for delivery rate as RHAU return to the colony only once a day to feed their chick (Gaston and Dechesne 1996), was low in 2012 compared to 2013, which corresponded to overall higher fledgling fCORT in 2012. This suggests that RHAU reduce their meal delivery rate and prioritize self‐maintenance over chick provisioning, a decision which may affect their young.

How adult RHAU foraging behavior (putatively aimed at maximizing foraging efficiency) mediates the effects of foraging conditions on chick nutritional stress is less clear. In other alcids, an increase in foraging effort is characterized by an increase in time spent under the water (Monaghan et al. 1994; Ronconi and Burger 2008; Young et al. 2015) and different diving patterns are associated with particular prey species (Elliott et al. 2008b). If that is the case, it may be argued that some prey, such as Japanese anchovy, require more effort to obtain than others as Teuri adults engaged in longer bouts and dove deeper than birds on St. Lazaria in both years (Table 3). However, in 2013, when fCORT in fledglings was generally low, birds on both colonies spent more time underwater and engaged in more foraging bouts, suggesting greater foraging effort. It is possible that when prey quality increases, RHAUs may be willing to expend more energy because the return on that energy is higher. Elliott et al. (2008a) found that the dive duration of thick‐billed murres (*Uria lomvia*) increased with prey mass. On St. Lazaria, RHAUs captured longer (2013 mean = 96.72 ± 1.03 [SE] mm; 2012 mean = 90.07 ± 0.8 mm) and heavier (2013 mean = 6.4 ± 0.2 g; 2012 mean = 3.99 ± 0.16 g) prey in 2013 compared to 2012, suggesting that prey size may explain the counterintuitive increase in the number and duration of bouts when foraging conditions appeared to be good.

Fluctuations in fCORT concentrations indicate that RHAU chicks can be affected by short‐term reductions in prey quality and changes in adult foraging behavior. Even brief exposure to elevated levels of CORT during development have been shown to result in changes in personality (Spencer and Verhulst 2007), an increased stress response (Kitaysky et al. 2001b; Love and Williams 2008), and reduced memory function (reviewed in Schoech et al. 2011; Kitaysky et al. 2006). Furthermore, short bouts of stress during development can result in reduced life expectancy (Haussmann et al. 2003; Boonekamp et al. 2014). Whether levels of stress associated with dietary changes affect postfledging survival and population processes at our focal colonies, however, remains to be examined.

The fCORT concentrations we observed in RHAU adults correlated with fCORT in fledglings, suggesting that adults are affected by dietary changes as well, perhaps due to reduced energy intake (adults and offspring feed on similar prey during chick rearing, Ito et al. 2009; Hipfner et al. 2013). While more direct evidence is needed, this correlation suggests that adults incur stress during the breeding season which carries over to the postbreeding molt, a circumstance that would hold true whether or not birds successfully bred. RHAUs undergo a simultaneous molt; they lose and regrow all of their flight feathers over a period of less than six weeks (Gaston and Dechesne 1996). During this time, their ability to capture prey is compromised and they may become vulnerable to food shortages and predation. To compensate for these drawbacks, many bird species are highly flexible in whether and when they commence feather loss and regrowth (Hahn et al. 1991). During postbreeding molt, RHAUs are no longer tied to their colony. Geolocators deployed on Teuri revealed that RHAU breeders rapidly relocated away from the colony to northern regions where autumn phytoplankton blooms provide a burst of productivity and abundant food resources (Takahashi et al. 2015). Potential flexibility in molt initiation and observations of postbreeding movement patterns suggest that adult fCORT (in this study, the feather tip, grown first) may be a signal of breeding season experiences rather than a measure of conditions at the molting location.

Our study provides some support for the junk‐food hypothesis and insight into how marine predators may be affected by changes in diet composition. We found that changes in diet composition occur at different temporal and spatial scales and clearly affected RHAUs. Chicks are responsive to minor changes in diet composition, adult foraging behavior is flexible, and adults may carry a signature of exposure to stress during the breeding season into the postreproductive period. This suggests that moderate changes in diet composition during the breeding season can affect seabird physiology and be detected in feathers. Thus, fCORT can be an effective way of assessing foraging conditions experienced during the chick‐rearing period. These findings also serve as a caution to the anthropogenic removal of forage fish biomass near seabird colonies (Cury et al. 2011; Essingtona et al. 2015) and illustrate that the effects of removal may not be detectable immediately with traditional monitoring methods (e.g., reproductive success or population trends). We conclude that RHAUs, a bird arguably adapted to variable environmental conditions, might be affected by relatively minor dietary changes and that foraging behavior and physiological responses to changes in prey composition should be considered when evaluating the effect of prey quality on seabirds and other marine predators.

## Data Accessibility

The data and R code from this research are available at the Dryad Digital Repository: http://dx.doi.org/10.5061/dryad.m8321.

## Conflict of Interest

The authors have no conflict of interests to declare.

## Supporting information


**Appendix S1** Methods and results of high‐performance liquid chromatography tandem mass spectrometry analysis of rhinoceros auklet adult and fledgling feathers for corticosterone.
