# Supplementary material for: Feather corticosterone reveals stress associated with dietary changes in a breeding seabird
Source: Ecol Evol. 2015 Sep 7;5(19):4221–32. doi: 10.1002/ece3.1694 (PMC4667832; doi:10.1002/ece3.1694)
Supplement: Supplementary file 1 — Appendix S1 Methods and results of high‐performance liquid chromatography tandem mass spectrometry analysis of rhinoceros auklet adult and fledgling feathers for corticosterone. [file ECE3-5-4221-s001.docx]

**Supplementary Information**

To identify whether rhinoceros auklet chicks and adults have corticosterone in their feathers we followed the high-performance liquid chromatography tandem mass-spectrometry (LC-APCI/MRM/MS) approach ([Koren *et al.* 2012](#_ENREF_2); [Keevil 2013](#_ENREF_1)).

**Methods**

Chemicals and Reagents

HPLC-grade standard corticosterone was purchased from Steroids Inc. (Newport, Rhode Island, USA). Deuterium labeled internal standard, corticosterone-2,2,4,6,6,17α,21,21-d8 (corticosterone-d8), was obtained from CDN Isotopes Inc. (Pointe-Claire, Quebec, Canada). HPLC-grade Methanol was purchased from Fisher Scientific (Edmonton, Alberta, Canada). Deionized water was prepared by Barnstead E-PURE Water System (Dubuque, Iowa, USA). LC-MS nitrogen was generated by a Source5000 nitrogen generator (Parker Balston, Haverville, Massachusetts, USA).

Sample Extraction Procedure

A standard solution or feather sample was placed in a 13 × 100 mm culture test tube, and 100 μL of deuterium labeled internal standard solution and 9 mL cold methanol were added. The test tube was capped and stored in a 4˚C fridge for 20 hours. After the feather was removed from the test tube, the extract was evaporated to dryness under nitrogen at 40˚C by use of Techne Sample Concentrator and reconstituted with 200 µL of acetonitrile:H_2_O (50/50, v/v). The sample was centrifuged at 14,000 rpm (Legend micro-21R, Thermo Scientific) for 20 minutes and 150 µL of its supernatant was submitted to LC-MS.

LC-APCI/MRM/MS Analysis

Extracts of two feather samples (one from an adult and one from a fledging) were analyzed by using an Agilent 1200 binary liquid chromatography (LC) system connected with an AB SCIEX QTRAP® 5500 tandem mass spectrometer equipped with an atmospheric pressure chemical ionization (APCI) source. LC separation was performed on an Agilent Poroshell 120 C18 column (50 x 3 mm, 2.7 µm particle size) at 45˚C. The mobile phase A was H_2_O/MeOH (75/25, v/v) and the mobile phase B was 100% methanol. The 8.5 min gradient was 20-50% B (0-1.0 min), 50-70% B (1.0-5.0 min), 70-100% B (5.0-5.5 min), 100-100% B (5.5-6.5 min), 100-20% B (6.5-7.0 min), and held at 20% B (7.0-8.5 min). The flow rate was 0.6 mL/min and the injection volume was 15 µL.

The analytes were ionized under positive APCI mode and the data were acquired via multiple reaction monitoring (MRM). Mass spectrometer conditions are listed in Table 1. Mass resolutions in Q1 and Q3 were set to unit resolution. Each analyte was monitored by two transitions (a quantifier and a qualifier) with conditions listed in Table 2.

Calibration Curve and Lower Limit of Quantification

Calibrant solutions were prepared by diluting the stock solution with water as shown in Table 3. Lower limit of quantification (LLOQ) of each steroid was defined by a statistical analysis.^[[1]](#footnote-1)^ Details of the testing results are available as an .xlsx file, on request.

**Table 1 Mass spectrometer conditions**

| **Parameter** | **Value** |
| --- | --- |
| Curtain gas | 35 psi |
| Temperature | 600 ˚C |
| Ion Source Gas 1 | 35 psi |
| Ion Source Gas 2 | 60 psi |
| Collision Gas | Medium |
| Nebulizer Current | 5 µA |

**Table 2 MRM conditions for the steroids analyzed in APCI positive mode. (DP= declustering potential, EP= entrance potential, CE= collision energy, and CXP= collision cell exit potential).**

| **Analyte** | **MRM**  **Transitions** | **DP**  **(V)** | **EP**  **(V)** | **CE**  **(eV)** | **CXP**  **(V)** |
| --- | --- | --- | --- | --- | --- |
| Corticosterone | 347/329  347/121 | 80 | 10 | 24 | 12 |
| Corticosterone-d8 | 355/337  355/125 | 80 | 10 | 24 | 12 |

**Table 3 Concentrations (ng/mL) of calibrators prepared in Methanol:H_2_O (50/50, v/v).^[[2]](#footnote-2)^**

| **Compound** | **STD 1** | **STD 2** | **STD 3** | **STD 4** | **STD 5** | **STD 6** | **STD 7** | **STD 8** |
| --- | --- | --- | --- | --- | --- | --- | --- | --- |
| Corticosterone | 20 | 10 | 4 | 2 | 1 | 0.4 | 0.2 | 0.1 |

**Table 4 Raw data acquired by QTRAP5500.**

| Sample Name | corticosterone (ng/mL) |
| --- | --- |
| Cal 8 | 0.12 |
| Cal 7 | 0.18 |
| Cal 6 | 0.40 |
| Cal 5 | 1.04 |
| Cal 4 | 1.79 |
| Cal 3 | 3.95 |
| Cal 2 | 9.36 |
| Cal 1 | 20.87 |
| Adult feather | 0.13 |
| Fledging feather | 0.21 |

**Results**

Corticosterone was detectable (intensity higher than LLOQ) in adult and fledgling feather tissues. Retention time was 2:51 (min:sec) and concentrations for the calibration curve and feather samples are reported in Table 4. Chromatograms for counts per second (cps) of the corticosterone ion are shown in Figures 1 (adult) and 2 (fledgling).


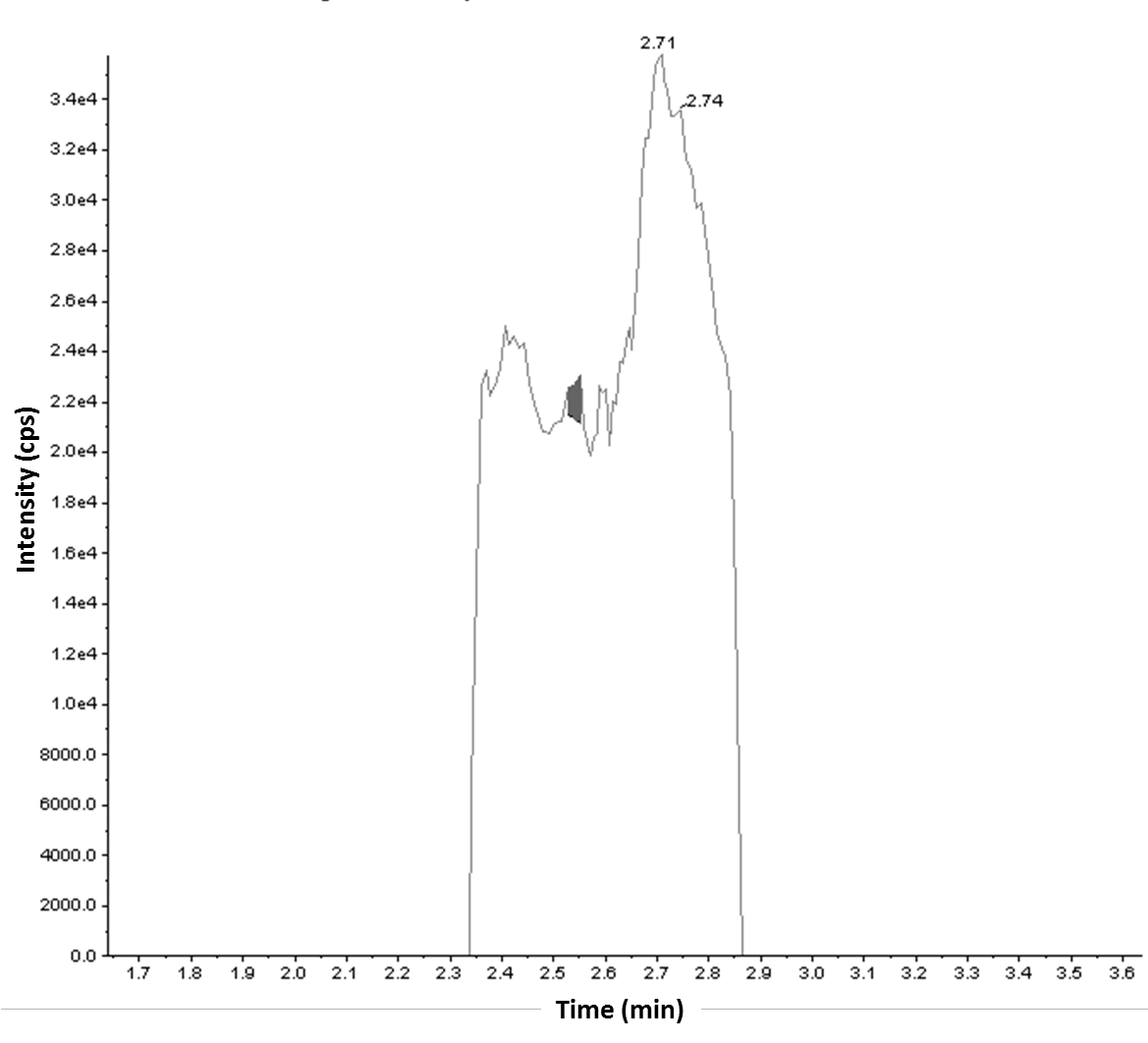


**Figure 1 Chromatogram for corticosterone concentration in the first primary feather of an adult rhinoceros auklet.** The shaded peak indicates the concentration of corticosterone present in the sample.


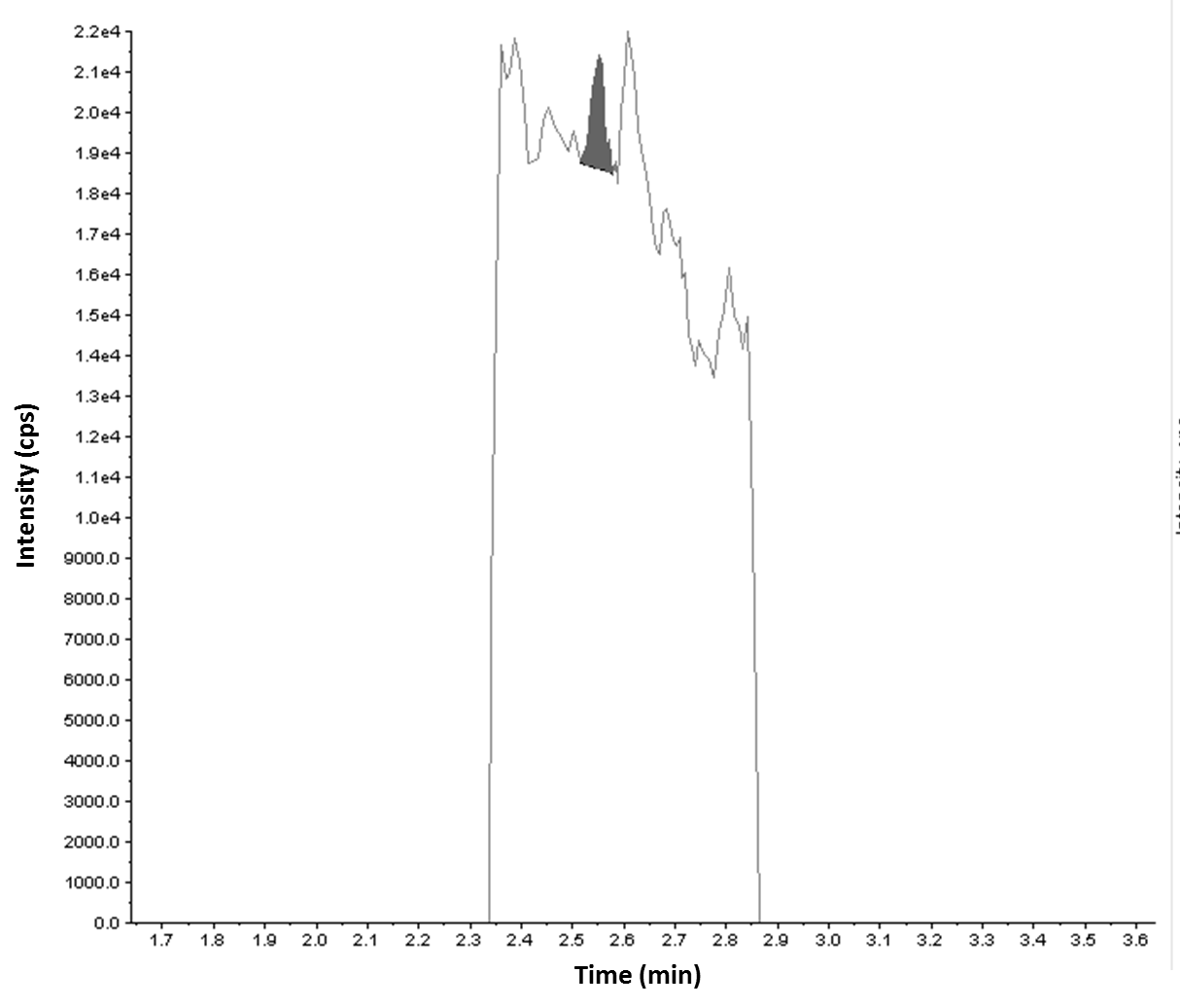


**Figure 2 Chromatogram for corticosterone concentration in the first primary feather of a rhinoceros auklet fledgling.** The shaded peak indicates the concentration of corticosterone present in the sample.

**References**

Keevil, B.G. (2013) Novel liquid chromatography tandem mass spectrometry (LC-MS/MS) methods for measuring steroids. *Best Practice & Research Clinical Endocrinology & Metabolism,* **27,** 663-674.

Koren, L., Nakagawa, S., Burke, T., Soma, K.K., Wynne-Edwards, K.E. & Geffen, E. (2012) Non-breeding feather concentrations of testosterone, corticosterone and cortisol are associated with subsequent survival in wild house sparrows. *Proc Biol Sci,* **279,** 1560-1566.

1. () <http://www.absciex.com/Documents/Downloads/Literature/mass-spectrometry-cms_059150.pdf> [↑](#footnote-ref-1)
2. () R^2^ of the calibration curve is greater than 0.99. [↑](#footnote-ref-2)
